# Identification of a Novel Alternatively Spliced Form of Inflammatory Regulator SWAP-70-Like Adapter of T Cells

**DOI:** 10.1155/2017/1324735

**Published:** 2017-04-24

**Authors:** Marie Hashimoto, Jun-ichi Nagao, Shojiro Ikezaki, Sonoko Tasaki, Ken-ichi Arita-Morioka, Yuka Narita, Tamaki Cho, Kenji Yuasa, Amnon Altman, Yoshihiko Tanaka

**Affiliations:** ^1^Section of Infection Biology, Department of Functional Bioscience, Fukuoka Dental College, Fukuoka 814-0193, Japan; ^2^Section of Image Diagnosis, Department of Diagnostics and General Care, Fukuoka Dental College, Fukuoka 814-0193, Japan; ^3^Advanced Science Research Center, Fukuoka Dental College, Fukuoka 814-0193, Japan; ^4^Division of Cell Biology, La Jolla Institute for Allergy and Immunology, La Jolla, CA 92037, USA

## Abstract

Activation of naive CD4^+^ T cells results in the development of several distinct subsets of effector Th cells, including Th2 cells that play a pivotal role in allergic inflammation and helminthic infections. SWAP-70-like adapter of T cells (SLAT), also known as Def6 or IBP, is a guanine nucleotide exchange factor for small GTPases, which regulates CD4^+^ T cell inflammatory responses by controlling Ca^2+^/NFAT signaling. In this study, we have identified a novel alternatively spliced isoform of SLAT, named SLAT2, which lacks the region encoded by exons 2–7 of the* Def6* gene. SLAT2 was selectively expressed in differentiated Th2 cells after the second round of in vitro stimulation, but not in differentiated Th1, Th17, or regulatory T (Treg) cells. Functional assays revealed that SLAT2 shared with SLAT the ability to enhance T cell receptor- (TCR-) mediated activation of NFAT and production of IL-4 but was unable to enhance TCR-induced adhesion to ICAM-1. Ectopic expression of SLAT2 or SLAT in Jurkat T cells resulted in the expression of distinct forms of filopodia, namely, short versus long ones, respectively. These results demonstrate that modulating either SLAT2 or SLAT protein expression could play critical roles in cytokine production and actin reorganization during inflammatory immune responses.

## 1. Introduction

During inflammatory immune responses under local cytokine environment, naive CD4^+^ T cells differentiate into distinct lineages including T helper 1 (Th1), Th2, Th17, and regulatory T (Treg) cells [1]. Both TCR-derived signals and the cytokine environment at the site of inflammatory immune responses play an important role in the fate decisions of Th subsets, and several TCR-proximal signaling intermediates can regulate Th subset differentiation [2]. Each Th cell subset expresses a unique set of transcription factors and produces hallmark cytokines [2], with Th2 cells being controlled by the master transcription factor, GATA3. Differentiated Th2 cells secrete IL-4, IL-5, and IL-13, which are important in inflammatory asthma and atopy, and constitute a defense against extracellular pathogens during inflammation. However, little information exists with regard to TCR-proximal signaling events that are unique to the differentiated Th2 subset.

We have previously isolated and characterized a TCR-regulated signaling protein termed SWAP-70-like adapter of T cells (SLAT) [3] encoded by the* Def6* gene and found it to share homology with SWAP-70, which is involved in B and mast cell activation [4–6] and to be expressed predominantly in Th cells and thymocytes. SLAT, also known as Def6 [7] or IBP [8], displays a guanine nucleotide exchange factor (GEF) activity for the Cdc42 and Rac1 small GTPases [9–14]. The human homolog of SLAT, termed IRF-4-binding protein (IBP), was independently isolated by another group [8]. Structurally, SLAT contains, beginning at its N terminus, a Ca^2+^-binding EF-hand domain [15], an immunoreceptor tyrosine-based activation motif- (ITAM-) like sequence, a phosphatidylinositol 3,4,5-trisphosphate-binding pleckstrin homology (PH) domain [3], and a catalytic Dbl homology (DH) domain [9–14, 16]. Our previous study of* Def6*-deficient mice revealed SLAT to be a critical selective regulator of the TCR-coupled Ca^2+^/NFAT signaling pathway, controlling positively CD4^+^ Th cell activation and differentiation, as evidenced by its critical role in the development of T cell-dependent inflammatory diseases such as allergic lung inflammation [17] and experimental autoimmune encephalomyelitis [18].

Alternative splicing is a critical mechanism that regulates gene expression at the posttranscriptional level. A number of genes exhibit significant changes in isoform expression during inflammatory immune responses, including several instances, in which alternative splicing changes are known to impact Th cell function [19]. Differential pre-mRNA splicing generates structural and functional diversities that contribute to various physiological processes such as differentiation into distinct lineages of Th cells.

In this study, we report the identification and characterization of a novel alternatively spliced isoform of SLAT, named SLAT2, which lacks the region encoded by exons 2–7 of the* Def6* gene. SLAT2 protein was expressed in differentiated Th2 cells after two rounds of in vitro stimulation, but not in differentiated Th1, Th17, and Treg cells. Similar to SLAT, SLAT2 enhanced TCR-mediated activation of NFAT and production of IL-4, but unlike SLAT [16] it was not required for TCR-induced adhesion to intercellular adhesion molecule-1 (ICAM-1). Ectopic expression of SLAT2 or SLAT in Jurkat T cells resulted in the expression of distinct forms of filopodia, namely, short versus long ones, respectively. Thus, SLAT2 in differentiated Th2 cells appears to exhibit distinct biological properties by comparison with SLAT. Differential expression of SLAT2 or SLAT protein could thus play critical roles in cytokine production and actin reorganization during inflammatory immune responses, leading to altered functional activities of these regulators.

## 2. Materials and Methods

### 2.1. Mice

OT-II TCR-transgenic mice (The Jackson Laboratory, Bar Harbor, ME) and C57BL/6N (B6) mice (Kyudo, Saga, Japan) were purchased and kept under specific pathogen-free conditions in the animal facility of Fukuoka Dental College. 6- to 8-week-old mice were used in all experiments. All experiments were performed in accordance with the guidelines of the committee of Ethics of Animal Experiments of Fukuoka Dental College.

### 2.2. Cell Culture and Stimulation

CD4^+^ T cells were purified from lymph node and spleen cells by magnetic sorting using Dynabeads Mouse CD4 followed by treatment with DETACHaBEAD Mouse CD4 (both from Invitrogen). Splenic antigen-presenting cells (APCs) from B6 mice were prepared by depletion of T cells through the use of CD90.2 MicroBeads Mouse (Miltenyi Biotec) and were treated with mitomycin C before use. CD4^+^ T cells (5 × 10^5^ per well) from OT-II or B6 mice were cultured in a 24-well plate with T cell-depleted, mitomycin C-treated B6 spleen cells (5 × 10^6^ per well) in a total volume of 2 mL in the presence of 1 *μ*g/mL ovalbumin (OVA)_323–339_ peptide or 1 *μ*g/mL anti-CD3 antibody (Ab) (145-2C11; BD), respectively. Th1 cells were cultured by the addition of 10 *μ*g/mL anti-IL-4 Ab (11B11; BD) and 10 U/mL recombinant murine IL-12 (Peprotech). Th2 cells were initiated by the addition of 10 *μ*g/mL anti-IL-12 Ab (C17.8; BD), 10 *μ*g/mL anti-IFN*γ* Ab (XMG1.2; BD), and 100 U/mL recombinant murine IL-4 (Peprotech). Th17 or Treg cells were differentiated in the presence of 2 ng/mL recombinant human TGF-*β*1 (R&D system) and 20 ng/mL recombinant murine IL-6 (Peprotech), or in the presence of 2 ng/mL recombinant human TGF-*β*1 and 2 ng/mL recombinant murine IL-2 (Peprotech), respectively. After 5 days, differentiated T cells subsets (1 × 10^6^ per well) were washed and restimulated using 1 *μ*g/mL anti-CD3 Ab- plus 1 *μ*g/mL anti-CD28 Ab (37.51; BD)-coated plates.

### 2.3. Cloning of the Gene Encoding SLAT2

CD4^+^ T cells from B6 mice were cultured under Th2-inducing conditions and then restimulated with plate-bound anti-CD3 Ab (1 *μ*g/mL) and anti-CD28 Ab (1 *μ*g/mL) for 2 days. The cells were harvested and total RNA was isolated using an RNeasy Kit (Qiagen). The RNA was reverse-transcribed into cDNA by oligo (dT) priming with the SuperScript III reverse transcriptase (Invitrogen). RT-PCR was performed with the primers 5′-ATGGCCCTGCGCAAGGAGCT-3′ and 5′-CTAATTCCCTGGTGCTGGAT-3′ and the amplified fragment having ~750 bp was cloned into the pGEM-T easy vector (Promega).

### 2.4. Plasmid Constructs

SLAT cDNA was cloned into the pEF-Myc-HisA vector (empty vector), resulting in the pEF-SLAT-Myc plasmid, as described previously [3]. SLAT2 cDNA was cloned into the* EcoRI* and* NotI* sites of pEF-Myc-HisA vector, giving rise to the pEF-SLAT2-Myc plasmid. A DNA fragment encoding the FLAG epitope (DYKDDDDK) was cloned into the* XhoI* and* XbaI* sites of pcDNA4/V5His-A, resulting in the pcDNA-FLAG plasmid. SLAT and SLAT2 cDNAs were PCR-amplified and cloned into the* SalI* and* NotI* sites of pcDNA-FLAG, fused in frame with the FLAG tag at their 3′ termini, yielding the plasmids pcDNA-SLAT-FLAG and pcDNA-SLAT2-FLAG, respectively. A PCR-amplified enhanced GFP (eGFP) gene was cloned into the* NheI* and* SalI* sites of the pSI vector to result in the plasmid pSI-EGFP. DNA fragments encoding SLAT-FLAG and SLAT2-FLAG were PCR-amplified from plasmids pcDNA-SLAT-FLAG and pcDNA-SLAT2-FLAG, respectively, and cloned into the* SalI* and* NotI* sites of pSI-EGFP, giving rise to the plasmids pSI-EGFP-SLAT-FLAG and pSI-EGFP-SLAT2-FLAG, respectively. SLAT cDNA was cloned into the retroviral vector pMIG, resulting in a pMIG-SLAT plasmid, as described previously [3]. SLAT2 cDNA was cloned into the* BglII* site of pMIG vector, resulting in plasmid pMIG-SLAT2.

### 2.5. Cell Transfection and Western Blotting Analysis

SV40 large T antigen-transfected human leukemic Jurkat T cells (Jurkat-TAg) in logarithmic growth phase were transfected with plasmid DNAs by electroporation using NEPA21 Super Electroporator (NEPAGENE, Japan). For detection of SLAT and SLAT2 proteins, the samples were separated by 11% SDS-PAGE, transferred to a PVDF membrane, and immunoblotted with anti-SLAT antisera prepared as described [3], anti-Myc Ab (9E10; Sigma), anti-GFP Ab (sc-9996; Santa Cruz Biotechnology), or anti-actin Ab (sc-1616; Santa Cruz Biotechnology). The signal intensity of SLAT and SLAT2 was quantified relative to the actin control using ImageJ software.

### 2.6. Luciferase Reporter Assay

Jurkat-TAg cells were transfected with an NFAT-luciferase plasmid plus a *β*-galactosidase (*β*-gal) reporter plasmid as described [20, 21]. After washing twice with phosphate-buffered saline (PBS), transfected cells were lysed in 100 *μ*L of lysis buffer (pH 7.8, 0.5% Triton X-100, 1 mM DTT, and 100 mM KPO_4_) for 10 min at room temperature. The lysate were centrifuged (12,000 ×g) for 10 min at 4°C. After centrifugation, 20 *μ*L of the supernatants was added with 50 *μ*L of Luciferase Assay Substrate (Promega). Luciferase activity was determined in a luminometer (GloMax 20/20 Single Tube Luminometer, Promega). Each group of luciferase activity was normalized to the cotransfected *β*-gal reporter activity.

### 2.7. Retroviral Transduction and Intracellular Cytokine Staining

Retroviral transduction was performed as described [22]. Briefly, Th2 cells were differentiated under Th2-inducing conditions by culture with T cell-depleted, mitomycin C-treated B6 spleen cells (5 × 10^6^) in the presence of 1 *μ*g/mL anti-CD3 for 5 days. The plasmids (10 *μ*g) were transfected into Platinum-E packaging cells using X-tremeGENE 9 DNA transfection reagent (Roche). The retroviral supernatants were harvested 48 h after transfection, supplemented with 4 ng/mL recombinant murine IL-2 (Peprotech) and 5 *μ*g/mL Polybrene (Sigma-Aldrich), and used to infect the differentiated Th2 cells that had been stimulated with plate-coated anti-CD3 plus -CD28 monoclonal antibodies (mAbs) (1 *μ*g/mL each) for 18 h after depletion of dead cells by Lympholyte-M (Cedarlane). After centrifugation for 1 h (800 ×g, room temperature), plates were incubated for 8 h at 32°C and for 16 h at 37°C. Two additional retroviral infections were carried out at daily intervals. One day after the last retroviral infection (day 4), IL-4 producing cells were analyzed by intracellular cytokine staining (ICS). ICS was performed as described [3]. Briefly, Th2 cells were restimulated with plate-bound anti-CD3 plus -CD28 mAbs (1 *μ*g/mL each) for 8 h in the presence of 10 *μ*g/mL brefeldin A (Sigma-Aldrich) during the final 2 h of culture. The stimulated cells were fixed in 3.7% paraformaldehyde (Wako) for 10 min at room temperature. The fixed cells were permeabilized with PBS containing 0.5% bovine serum albumin (BSA) plus 0.5% saponin (Sigma-Aldrich) and stained with PE-conjugated anti-IL-4 Ab (11B11; BD) for 30 min. After washing twice with PBS containing 0.5% BSA and 0.5% saponin, the stained cells were analyzed using a FACSCalibur (BD). Data were analyzed with FlowJo software.

### 2.8. Adhesion Assay

Adhesion to ICAM-1 was performed as described [16] with slight modification. Briefly, 5 *μ*g/mL goat anti-human-IgG (Fc) Ab (Jackson ImmunoResearch)-immobilized plates were coated with 1 *μ*g/mL fusion protein (Fc-ICAM-1) of recombinant mouse ICAM-1 and human IgG1 Fc fragment (R&D Systems) overnight at 4°C. The coated plates were blocked with 1% BSA in PBS for 1 h at room temperature. Transfected cells were labeled with 2.5 *μ*M Calcein-AM (DOJINDO) for 30 min at 37°C in Phenol-Red-free Hanks' balanced salt solution, washed twice, and incubated (1 × 10^5^) on Fc-ICAM-1-coated plates, in the absence or presence of 10 *μ*g/mL anti-CD3 Ab (OKT3; Biolegend). Obtained specific adhesion was expressed as a percentage of output fluorescence divided by input fluorescence.

### 2.9. Confocal Microscopy

Transfected Jurkat-TAg cells were placed on poly-L-lysine-coated coverslips with or without 2 *μ*g/mL anti-CD3 Ab (OKT3; Biolegend) for 30 min at 37°C, fixed in 4% paraformaldehyde for 10 min at room temperature, and stained with Alexa Fluor 546 phalloidin (Thermo Fisher Scientific) and 4′,6′-diamidino-2-phenylindole (DAPI) (DOJINDO) for 40 min at room temperature. Images were captured with a LSM710 confocal laser scanning microscope (Carl Zeiss, Germany) with a 100x/1.40 Oil DIC objective.

### 2.10. Statistical Analysis

Statistical analysis was performed by using two-tailed Student's* t*-test.

## 3. Results

### 3.1. Identification of a Novel SLAT Splice Variant

**Figure 1:**
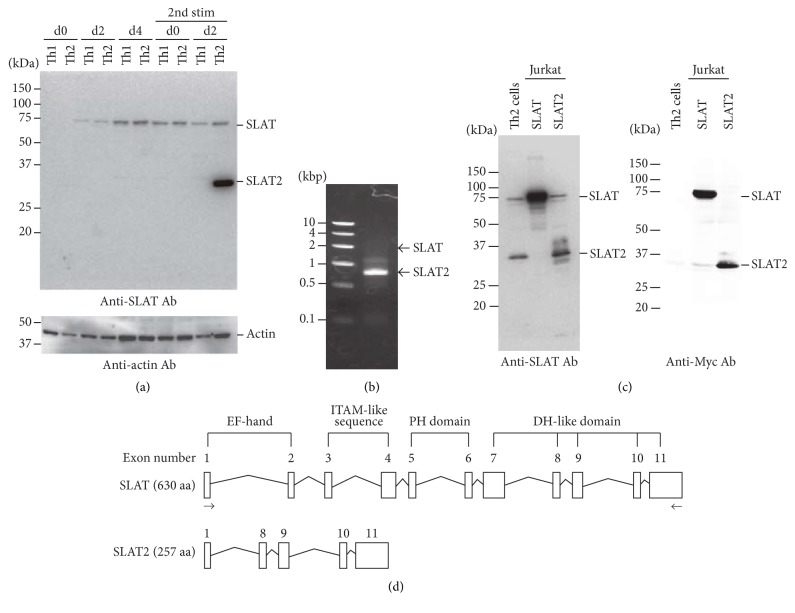
Identification of SLAT2, a novel SLAT splice variant. (a) CD4^+^ T cells from B6 mice were differentiated under Th1- or Th2-inducing conditions with spleen-derived APCs and anti-CD3 Ab for 5 days, and the cells were then restimulated with plate-bound anti-CD3 plus -CD28 mAbs. Cell lysates were prepared at the indicated days and analyzed by Western blotting using anti-SLAT Ab. Data are representative of three independent experiments. (b) Gel electrophoresis of RT-PCR products amplified from Th2 cells two days after the second round of stimulation using primers spanning the entire SLAT open reading frame. Arrows indicate the position of SLAT2 (lower) or a predictable full length SLAT (upper). (c) Expression of SLAT and SLAT2 proteins in differentiated Th2 cells and Jurkat-TAg cells transfected with pEF-SLAT or pEF-SLAT2. Differentiated Th2 cells from B6 mice were restimulated with plate-bound anti-CD3 plus -CD28 mAbs for 4 days. Cell lysates were analyzed by anti-SLAT or anti-Myc immunoblotting. Data are representative of three independent experiments. (d) Genomic structure of SLAT and the alternatively spliced isoform SLAT2. Primers used for PCR amplification are indicated with arrows. Exons and introns are shown as open boxes and lines, respectively. SLAT2 contains the DH-like domain but lacks the part of EF-hand and the entire ITAM-like sequence and PH domain.

To examine the expression of SLAT during Th2 differentiation, B6 naive CD4^+^ T cells were activated in vitro under Th2-inducing conditions. Western blotting analysis using an anti-SLAT Ab revealed an unexpected smaller size SLAT-related molecule in differentiated Th2 cells, but not in differentiated Th1 cells, two days after the second round of in vitro stimulation (Figure 1(a)). To better characterize this gene product, we performed an RT-PCR on cDNA derived from Th2 cells two days after the second round of stimulation using primers designed to amplify the entire SLAT open reading frame (ORF). This resulted in amplification of a ~750 bp product revealed after a short (10 s) extension reaction, at which time the PCR product corresponding to the full length SLAT cDNA was not evident (Figure 1(b)). The ~750 bp PCR product was subcloned and sequenced, revealing a previously unreported* Def6* ORF, in which exons 2–7 were absent and exon 1 was directly spliced to exon 8 without frameshifts. This ORF potentially encodes a mature peptide comprising 257 amino acids. Western blotting analysis revealed that Jurkat-TAg cells transfected with the alternatively spliced SLAT cDNA expressed, as expected, a protein with a molecular mass of ~35 kDa, designated as SLAT2, in primary differentiated Th2 cells after the second round of stimulation. In contrast, SLAT-transfected cells expressed a protein of ~75 kDa (Figure 1(c)). Residues 33–257 are identical to the DH domain of SLAT. Primary structures of SLAT and SLAT2 are shown in Figure 1(d).

### 3.2. Expression and Regulation of SLAT2

**Figure 2:**
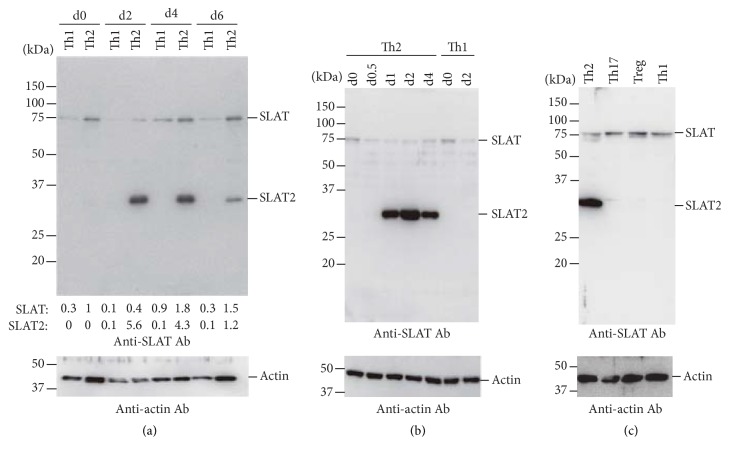
Kinetics of SLAT2 expression in differentiated Th cells. (a) CD4^+^ T cells from B6 mice were differentiated under Th1- and Th2-inducing conditions as in Figure 1. Cell lysates were prepared at the indicated days and analyzed by Western blotting using anti-SLAT Ab. Numbers under the SLAT blot indicate intensity of SLAT and SLAT2 signals relative to SLAT signal of Th2 cells on day 0 (=1) after normalization to the actin signal. (b) CD4^+^ T cells from OT-II mice were differentiated under Th1- or Th2-inducing conditions with splenic APCs and 1 *μ*g/mL OVA_323–339_ peptide for 5 days, and the cells were further restimulated with plate-bound anti-CD3 plus -CD28 mAbs. Cell lysates were prepared at the indicated days and analyzed as in (a). (c) CD4^+^ T cells from B6 mice were differentiated into the indicated Th cell subset and restimulated with splenic APCs and anti-CD3 Ab for 4 days. Cell lysates were analyzed by Western blotting as in (a). Data are representative of at least two independent experiments.

To determine the kinetics of SLAT2 protein expression, B6 naive CD4^+^ T cells differentiated under Th2- or Th1-inducing conditions were restimulated on day 5 with plate-coated anti-CD3 plus -CD28 mAbs under neutral culture conditions (Figure 2(a)). Prior to restimulation (day 0), SLAT2 was not expressed in either Th2 or Th1 cells, while SLAT was expressed in Th2, but not (or substantially less) in Th1 cells. Importantly, restimulation led to a substantial upregulation of SLAT2 protein expression on day 2 in Th2 cells, but not in Th1 cells. This upregulation was sustained on day 4 and decreased by day 6. In contrast, upregulation of SLAT was not observed on day 2 but was evident on days 4 and 6 in Th2 cells. To evaluate the expression of SLAT2 and SLAT under conditions of specific antigen stimulation, we obtained naive CD4^+^ T cells from OT-II TCR-transgenic mice, which express an OVA-specific TCR, and cultured the cells with a low dose of OVA peptide and splenic APCs from B6 mice under Th2- or Th1-inducing conditions. Similar to B6 Th cells, SLAT2 was not expressed during the initial round of OVA stimulation (data not shown). However, following the second round of stimulation, the expression of SLAT2 was induced within one day reaching a maximum on day 2, with no SLAT2 detected after 0.5 days of stimulation (Figure 2(b)). Next, we examined the expression of SLAT2 in other subsets of differentiated Th cells, namely, Th17 and Treg cells. SLAT2 was observed in restimulated Th2 cells, but not in differentiated Th1, Th17, or Treg cells (Figure 2(c)). In contrast, SLAT was expressed in all subsets of differentiated Th cells. These results indicate that the expression of SLAT2 in differentiated and restimulated T cells is unique to the Th2 subset.

### 3.3. SLAT2 Enhances TCR-Induced NFAT Activity and IL-4 Expression

**Figure 3:**
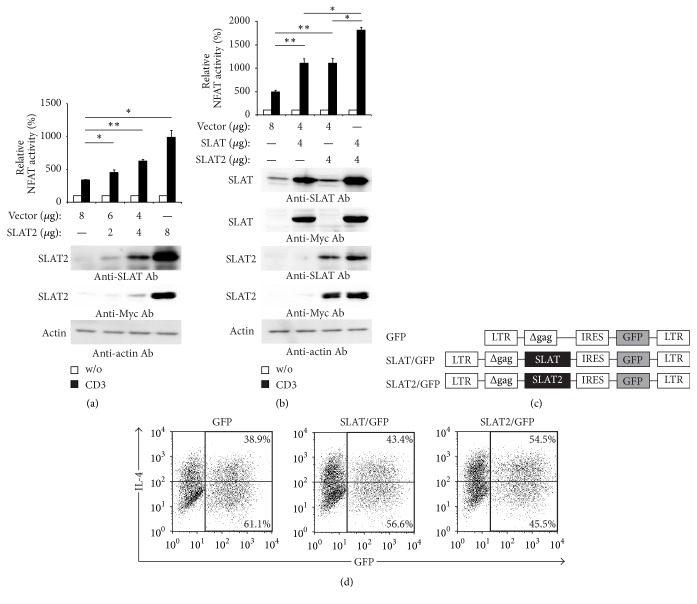
SLAT2 enhances TCR-induced NFAT activity and IL-4 expression. (a and b) Jurkat-TAg cells (5 × 10^6^) were cotransfected with the indicated plasmid cDNA amounts of empty vector or SLAT and/or SLAT2 together with NFAT-Luc (5 *μ*g) and *β*-galactosidase (*β*-gal) (5 *μ*g) reporter plasmids. The cells were left unstimulated or were stimulated with 2 *μ*g/mL anti-CD3 Ab (OKT3) for 6 h at 37°C. Luciferase activity was normalized to the activity of *β*-gal and data are displayed as % NFAT activity relative to basal activity in unstimulated cells (=100%). Data represent means of triplicates, and graphs show means ± SD. Statistical analysis was performed using Student's* t*-test (^*∗*^*P* < 0.01; ^*∗∗*^*P* < 0.001). Expression of transfected SLAT and SLAT2 or endogenous actin proteins was detected by Western blotting (bottom panels). Data are representative of three independent experiments. (c) Schematic diagram of retroviral SLAT and SLAT2 constructs in the pMIG vector. (d) Five-day differentiated Th2 cells were restimulated with anti-CD3 plus -CD28 mAbs and infected with the indicated retroviruses. Cells were harvested on day 9 (4 days after second stimulation), restimulated with anti-CD3 plus -CD28 mAbs, and IL-4-producing cells were enumerated by intracellular cytokine staining. Data are representative of two independent experiments.

SLAT promotes Th1 and Th2 cell differentiation by controlling NFAT activation in CD4^+^ T cells [17] and enhances TCR-induced NFAT activity [11]. To understand the function of SLAT2, we examined the effect of ectopic SLAT2 versus SLAT expression on the TCR-induced activation of an NFAT-luciferase reporter gene in Jurkat-TAg cells. Similar to SLAT and as previously shown [11], SLAT2 induced a dose-dependent increase in NFAT activity relative to empty vector-transfected cells (Figure 3(a)). Thus, SLAT2 can enhance the TCR-induced NFAT activity, just like SLAT. Next, we determined whether additional SLAT2 expression enhanced SLAT-mediated NFAT activity under TCR stimulation. Importantly, SLAT2 had an additive effect combined with SLAT on the TCR-induced NFAT activity (Figure 3(b)). These results suggest that the activation of TCR-induced NFAT is enhanced by SLAT2 and/or SLAT in Jurkat T cells.

NFAT activation promotes the expression of IL-4 in Th2 cells [23, 24]. To explore whether SLAT2 can regulate the production of IL-4, we generated a retroviral SLAT2 expression vector (Figure 3(c)), which was used to infect differentiated Th2 cells, resulting in a transduction efficiency of 25%–45% (data not shown). We have previously shown that transduction of differentiated Th2 cells with SLAT increases the fraction of IL-4-producing cells in the GFP^+^ population [3]. Importantly, under the same conditions SLAT2 increased the fraction of IL-4-producing cells,* that is*, from ~39% to ~55% IL-4^+^ cells (Figure 3(d)), even though SLAT minimally increased the fraction of IL-4-producing cells from ~39% to ~43% (Figure 3(d)). These results indicate that SLAT2 increases the level of IL-4-producing cells by differentiated Th2 cells.

### 3.4. SLAT2 Is Not Required for TCR-Induced Adhesion to ICAM-1

**Figure 4:**
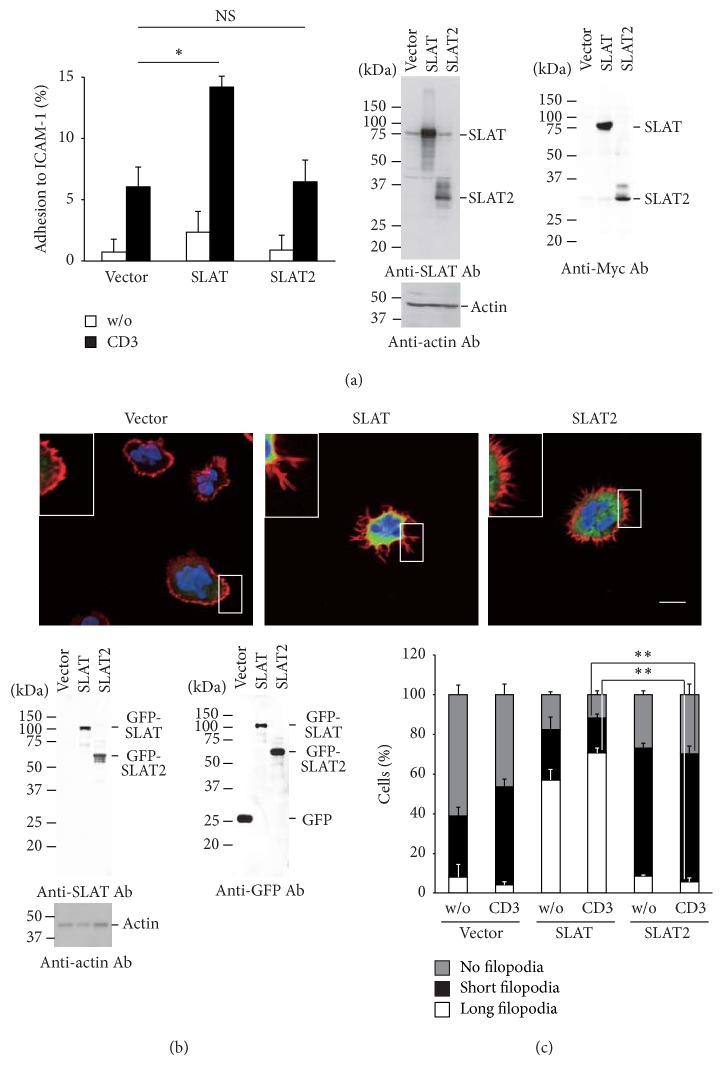
Effect of SLAT2 expression on T cell adhesion and filopodia formation. (a) Jurkat-TAg cells (5 × 10^6^) were transfected with empty vector, pEF-SLAT or pEF-SLAT2 (8 *μ*g each). The cells were either left unstimulated or stimulated with 10 *μ*g/mL anti-CD3 Ab (OKT3) for 45 min and subsequently analyzed for adhesion to plate-bound Fc-ICAM-1. Data represent means of triplicates, and graphs show means ± SD (left). Expression of transfected proteins was detected with an anti-SLAT Ab or anti-Myc Ab (right). Statistical analysis was performed using Student's* t*-test (^*∗*^*P* < 0.01; NS, not significant). (b) Jurkat-TAg cells (5 × 10^6^) were transfected with pSI-EGFP vector, pSI-EGFP-SLAT, or pSI-EGFP-SLAT2 (8 *μ*g each). The cells were stimulated with 2 *μ*g/mL anti-CD3 Ab (OKT3), immobilized on poly-L-lysine-coated coverslips, and fixed and stained with Alexa Fluor 546 Phalloidin and DAPI. Representative confocal images of stimulated cells imaged in each group are shown (upper). Scale bar, 10 *μ*m. Expression of transfected proteins was detected with an anti-SLAT Ab or anti-GFP Ab (lower). (c) Quantitative analysis of the imaging data in panel (b). Cells were categorized into three groups, namely, long filopodia, short filopodia, or no filopodia. Long filopodia are defined as having a length ~5 times longer than short filopodia [43]. The percentage of cells in each class is shown. >100 cells were counted in each group. Data represent means of triplicates, and graphs show means ± SD. Statistical analysis was performed using Student's* t*-test (^*∗∗*^*P* < 0.001). Scale bar, 10 *μ*m. Data are representative of three independent experiments.

SLAT promotes the TCR-induced adhesion of T cells to ICAM-1 via leukocyte function-associated antigen-1 (LFA-1) activation [16]. To explore the role of SLAT2 in regulating LFA-1 function following TCR engagement, we examined the effect of ectopic SLAT2 expression on the TCR-mediated adhesion to ICAM-1. SLAT-transfected Jurkat-TAg cells showed an increase in TCR-induced adhesion to ICAM-1 relative to control transfectants, as previously shown [16]. In contrast, SLAT2 had no effect on TCR-induced adhesion to ICAM-1 (Figure 4(a)). Thus, unlike SLAT, SLAT2 does not appear to enhance TCR-induced adhesion to ICAM-1 in T cells. These results suggest that SLAT2 may function in a manner different from SLAT during TCR-mediated T cell activation.

### 3.5. SLAT2 Localizes within Cytoplasm and Induces Short Filopodia

It is known that SLAT localizes to the plasma membrane and spreads diffusely throughout the cytoplasm in COS-7 cells [10, 13, 25]. To determine the cellular distribution of SLAT2, GFP-tagged SLAT2 or SLAT was expressed with Alexa Fluor 546-labeled phalloidin in Jurkat-TAg cells. The intracellular location of the proteins was determined by confocal microscopy. GFP-SLAT was found to colocalize with F-actin at the plasma membrane with or without TCR stimulation and, furthermore, compared to the empty vector-transfected cells, GFP-SLAT expression resulted in an apparent formation of long filopodia in Jurkat T cells in unstimulated T cells, and anti-CD3 stimulation caused a further increase (Figure 4(b), middle panel). In contrast, GFP-SLAT2 localized within the cytoplasm but did not colocalize with F-actin near the plasma membrane. Furthermore, GFP-SLAT2 mostly induced short filopodia formation in Jurkat T cells in either the absence or presence of TCR stimulation (Figure 4(b), right panel). In the control cells, faint short filopodia formation was observed in some GFP-transfected Jurkat-TAg cells. Quantitation of filopodia formation in the transfected T cells (Figure 4(c)) revealed that ectopic GFP-SLAT2 protein increased short filopodia formation in TCR-stimulated cells (17.3% ± 2.1% in GFP-SLAT-transfected cells versus 64.7% ± 3.5% in GFP-SLAT2-transfected cells; *P* < 0.001). In contrast to SLAT2, GFP-SLAT preferentially mediated long filopodia formation (70.7% ± 2.5% in GFP-SLAT-transfected cells versus 5.7% ± 2.1% in GFP-SLAT2-transfected cells; *P* < 0.001). These results indicate that, unlike SLAT, SLAT2 expression augments short filopodia formation at the expense of long filopodia in stimulated T cells.

## 4. Discussion

Alternative splicing is an important mechanism for controlling gene expression and increasing protein diversity from a single primary transcript. In the immune system, alternative splicing allows large proteomic complexity from a limited number of genes in inflammatory immune regulation network. Indeed, a few recent notable examples of alternative splicing, in CD4^+^ T cell-expressed mucosa-associated lymphoid tissue protein 1 (MALT1) [26] and Foxp3 [27], have been demonstrated to regulate T cell responses to antigen [19]. In the present study, we have identified a new alternatively spliced product, termed SLAT2, of the murine* Def6* gene in differentiated Th2 cells after the second round of stimulation. SLAT2 mRNA is generated by the splicing of exons 2–7 that results in a partial deletion of Ca^2+^-binding EF-hand and in the complete deletion of the ITAM-like sequence and the PH domain, generating a correct reading frame and a translated product that contains the C-terminal DH domain. The selective and transient upregulation of SLAT2 induced in differentiated Th2 cells by TCR restimulation suggests that SLAT2 may play a role in Th2-mediated immune responses,* for example*, allergic lung inflammation.

NFAT, a major regulator of T cell activation, is critical for IL-4 production and Th2 differentiation [23, 24]. Increasing evidence indicates that SLAT, which is predominantly expressed in T cells, plays important roles in TCR-mediated signaling by linking actin cytoskeleton reorganization and Cdc42 activation to the Ca^2+^-NFAT signaling pathway and consequently to Th cell differentiation during inflammation [11, 12, 17, 28]. Our recent study demonstrated that the N-terminal EF-hand domain and the PH domain of SLAT interact with inositol 1,4,5-triphosphate receptor type 1 and that this interaction is important for TCR-mediated Ca^2+^-NFAT signaling [15]. Interestingly, SLAT2 still possessed the ability to enhance NFAT activation, and coexpression of SLAT2 with SLAT resulted in further enhancement of TCR-induced NFAT activation despite the fact that SLAT2 lacks the ITAM-like sequence and PH domain, which were previously shown to contribute to the Ca^2+^-NFAT signaling function of SLAT [11]. In addition, confocal microscopy analysis showed that SLAT2 did not translocate to the plasma membrane upon TCR stimulation. We therefore propose that cytosolic SLAT2 can enhance Ca^2+^-NFAT signaling in a different way from SLAT or it may be able to act additively to SLAT in this pathway. We have also provided evidence that ectopic SLAT2 expression enhances the production of IL-4, in differentiated Th2 cells. Thus, the enhancement of NFAT activation induced by SLAT2 could account for the increased IL-4 expression by SLAT2-transduced Th2 cells.

SLAT2 expression was transiently and selectively upregulated in differentiated Th2 cells within one day of the second TCR stimulation, reached a maximum after two days, and declined by the sixth day, even though upregulation of SLAT2 was not observed during the first stimulation at all. SLAT2 is therefore expressed at a relatively late stage of Th cell differentiation, suggesting that it may not contribute to early Th2 cell differentiation per se, but may function during the effector phase of Th2 cell function. Differentiated Th2 cells transcribe* Il4* as early as 1 day after TCR restimulation (data not shown), a time when SLAT2 was already substantially upregulated. Further work should be aimed at defining the precise mechanisms that regulate the enhanced IL-4 expression by SLAT2 in differentiated Th2 cells, including in vivo, during inflammation.

Some of SLAT functions were not observed in SLAT2, including adhesion to ICAM-1 that reflects LFA-1 activation, and long filopodia formation in Jurkat T cell. SLAT is involved in TCR-induced adhesion through interaction of its PH domain with Rap1 GTPase, which was required for T cell adhesion to ICAM-1 [16]. Therefore, the deletion of the PH domain in SLAT2 likely explains the inability of SLAT2 to promote adhesion to ICAM-1. Thus, the functions of SLAT2 seem to be distinct from those of SLAT in TCR-mediated activation. SLAT is expressed and functions not only in Th cells but also in other types of cells, such as macrophages [29, 30], osteoclasts [31], and cancer cells [32–35]. Thus, it would be interesting to determine whether SLAT2 is expressed and functions in these other cell types.

Cdc42 member of the Rho family of small GTPases is cytosol in its GDP-bound resting form. Cdc42 functions as a molecular switch by cycling between GDP-bound inactive and GTP-bound active states and is recruited to membranes where its GDP is exchanged for GTP through the action of several GEFs with known reactivity toward Rho family GTPases [36]. Dbl family proteins, which possess a catalytic DH domain followed by a PH domain, constitute the largest subgroup of Rho-specific GEFs, followed by the dedicator of cytokinesis (DOCK) family, whose members possess a DOCK homology region 2 (DHR2) instead of a DH domain. The smallest subgroup of SWAP-70 subfamily includes SWAP-70 and SLAT, which contain a DH-like domain but neither a DH nor a DHR2 domain. Some of these Rho GEFs,* that is*, Vav [22] and *β*PIX [37] (Dbl subfamily), DOCK8 [38] (DOCK subfamily), and SLAT [3, 9–14, 39–42] (SWAP-70 subfamily), participate in TCR signaling and Th cell differentiation and are known to mediate GEF activity on Cdc42. The SLAT DH-like domain possesses TCR-mediated, plasma membrane-residing GEF activity toward Cdc42 that controls dynamics of the actin cytoskeleton and leads to long filopodia formation [10]. Additionally, the TCR-induced tyrosine phosphorylation of the ITAM-like sequence in SLAT is required for its translocation to the plasma membrane and immunological synapse [11]. We observed lack of membrane localization of SLAT2, which correlated with reduced long filopodia formation in SLAT2-transfected T cells compared to SLAT-transfected cells. Instead, SLAT2 increased formation of short, rather than long, filopodia in TCR-stimulated cells. Therefore, the differential localization and function of the two SLAT proteins and, potentially, differences in their reactivity toward Cdc42 might play an important role in regulating the formation of filopodia at the site of inflammatory immune responses.

## 5. Conclusions

Collectively, our results suggest that alternative splicing of SLAT may exhibit differential biological activities upon TCR-mediated stimulation and that modulating either SLAT2 or SLAT protein expression could play critical roles in cytokine production and actin reorganization during inflammatory immune responses, leading to altered functional activities of these regulators. Precise understanding of the mechanism through which SLAT2 modulates TCR-mediated signaling and Th2 cell function awaits future studies.
